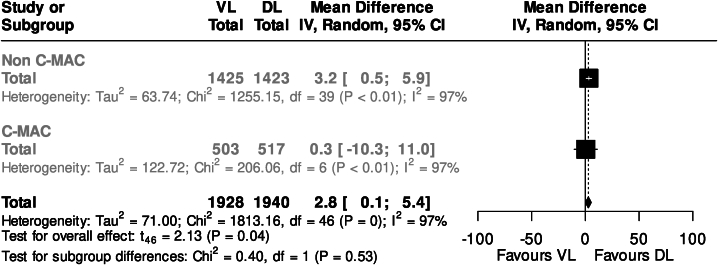# Videolaryngoscopy versus direct laryngoscopy for paediatric tracheal intubation: a systematic review with meta-analysis and trial sequential analysis

**DOI:** 10.1016/j.bja.2025.07.094

**Published:** 2025-10-03

**Authors:** Gabriela Koepp-Medina, Andrea C. Lusardi, Benedetta Di Fonzo, Markus Huber, Arash Afshari, Rachele Bonfiglio, Lea Zimmermann, Vera Bohnenblust, Robert Greif, Janne Vendt, Thomas Riva, Nicola Disma, Alexander Fuchs

**Affiliations:** 1 Department of Anaesthesiology and Pain Medicine, Inselspital, Bern University Hospital, University of Bern, Bern, Switzerland; 2 Unit for Research in Anaesthesia, IRCCS Istituto Giannina Gaslini, Genoa, Italy; 3 Department of Paediatric and Obstetric Anaesthesia, Juliane Marie Centre, Rigshospitalet & Institute of Clinical Medicine, University of Copenhagen, Copenhagen, Denmark; 4 Department of Surgical Science, University of Turin, Turin, Italy; 5 Faculty of Medicine, University of Bern, Bern, Switzerland; 6 Medical Library Rigshospitalet, University of Copenhagen, Copenhagen, Denmark

**Keywords:** airway, direct laryngoscopy, intubation, paediatric anaesthesia, videolaryngoscopy

## Abstract

**Background:**

Paediatric tracheal intubation presents unique challenges owing to anatomical and physiological characteristics. Multiple intubation attempts increase the risk of severe complications; therefore, achieving high first-pass success is critical. Videolaryngoscopy (VL) represents an alternative to direct laryngoscopy (DL), but its comparative efficacy, safety, and clinical impact in paediatric practice remain under investigation.

**Methods:**

This systematic review and meta-analysis evaluated VL *vs* DL for paediatric tracheal intubation, focusing on first-attempt success rate, glottic visualisation, intubation time, and critical events. Eight databases were searched. RCTs comparing VL and DL in paediatric patients (<16 yr) were included.

**Results:**

Of 22 221 screened articles, 53 RCTs (4887 patients) met inclusion criteria. VL and DL showed comparable first-attempt tracheal intubation success rates, without a statistically significant difference (risk ratio: 1.03, 95% confidence interval [95% CI]: 0.99–1.07, *P*=0.18). Certainty of evidence was rated as high by the Grading of Recommendations Assessment, Development, and Evaluation criteria. Intubation time was slightly longer with VL (mean difference: +3 s, 95% CI: 0.1–5.4). VL improved glottic visualisation across all paediatric age groups (Percentage of Glottic Opening score, mean difference: 9.8%, 95% CI: 3.2–16.4, *P*<0.01) and reduced oesophageal intubation in children under 1 yr of age (risk ratio: 0.16, 95% CI: 0.06–0.40, *P*<0.01).

**Conclusions:**

In paediatric patients with mostly normal airways, no significant differences were observed between VL and DL in achieving first-attempt tracheal intubation success. VL improved glottic visualisation, and, in patients under 1 yr of age, reduced oesophageal intubation with low certainty of evidence. Trial sequential analysis indicates that the available data remain underpowered to confirm a definitive effect.

**Systematic review protocol:**

PROSPERO (CRD42024498524).

**Editor’s key points**

• Tracheal intubation in children can be challenging, and multiple attempts increase the risk of complications. The comparative effectiveness of videolaryngoscopy *vs* direct laryngoscopy in paediatric patients remains uncertain.
• This systematic review and meta-analysis of 53 RCTs found no significant difference in first-attempt tracheal intubation success between the techniques in children with mostly normal airways. Videolaryngoscopy improved glottic visualisation, and, in infants under 1 yr, reduced oesophageal intubation.
• Evidence remains insufficient to confirm definitive benefits, particularly in children with difficult airways.

Paediatric and neonatal tracheal intubation is an essential skill for airway providers in various clinical settings, including anaesthesia, emergency medicine, and intensive care.^1^ Direct laryngoscopy (DL) allows the visualisation of glottic structures and has been traditionally considered the standard of care for paediatric tracheal intubation.^2^

Compared with adults, children have a more cephalad larynx; a relatively larger head and tongue; a narrow, floppy, u-shaped epiglottis; and a smaller mouth opening.^3^ These characteristics present specific difficulties for laryngoscopy and intubation.^4^ Additionally to anatomical characteristics, physiological factors in children pose challenges for airway management providers. The physiologically difficult airway in children results from their higher oxygen consumption, lower functional residual capacity, and a higher closing capacity. Multiple intubation attempts and unsuccessful tracheal intubations amplify the risks of hypoxaemia, which ultimately can lead to hypoxic encephalopathy, cardiac arrest, or death.^5^, ^6^, ^7^ For this reason, the airway management guidelines in neonates and infants suggest (a) limiting tracheal intubation attempts to a maximum of four (one by a physician in training and three by a senior anaesthesiologist), (b) reassessment and a change in technique or provider after each attempt, and (c) consideration of waking the patient if the airway cannot be secured or (d) performing emergency front-of-neck access (eFONA)^8^ in case of impaired oxygenation.^9^ Achieving first-attempt success in paediatric tracheal intubation is crucial owing to the limited margin for error.^10^

Videolaryngoscopy (VL) has become more widely available, and recently randomised studies suggested that VL improves the visualisation of the glottis and success rates and reduces complications in children with normal airway or with known or unanticipated difficult intubation compared with DL.^11^^,^^12^

However, the comparative efficacy of VL *vs* DL for paediatric tracheal intubation remains a subject of ongoing debate and investigation.^13^ Therefore, to provide an update of the available evidence, we conducted a systematic review and meta-analysis to compare VL *vs* DL for paediatric tracheal intubation regarding effectiveness in first-attempt intubation success rate, visualisation of the glottis, time to intubation, and incidence of critical events.

## Methods

The protocol of this systematic review and meta-analysis was registered prospectively at PROSPERO (CRD42024498524) on February 21, 2024. We report the findings according to the Preferred Reporting Items for Systematic Reviews and Meta-Analyses (PRISMA) guidelines.^14^

### Eligibility criteria

This review included peer-reviewed published RCTs in paediatric patients (<16 yr of age) requiring orotracheal intubation in elective or emergency in-hospital settings. Eligible studies compared VL with DL. VL was defined as the use of a video laryngoscope equipped with a camera at the tip of the blade to display the vocal cords on either an attached or external screen. Unpublished studies, case series, conference abstracts, trial protocols, duplicates, and unavailable articles were excluded. There were no restrictions regarding language.

### Searched databases and search strategy

To identify potentially relevant publications on the topic, a search strategy was designed, and a search was performed in MEDLINE, Embase, Cochrane Library, CINAHL, Web of Science Core Collection, Scopus, ClinicalTrials.gov, and ICTRP (WHO).

A medical information specialist (JV) developed the search strategy and tested it against a list of core references to ensure key publications were included. After refinement, the information specialist set up the search strategy for each information source based on database-specific controlled vocabulary (index terms) and free text. The free text search included synonyms, acronyms, and similar terms. No database-provided limits have been applied in any sources considering study types, languages. The search was conducted and updated to cover the period from 2000 to January 3, 2025, before the data extraction. The detailed search is available in the Supplementary material A.

### Study selection

After identifying relevant publications, all were imported into Covidence (Veritas Health Innovation, Melbourne, Australia)^15^ for deduplication. Rotating pairs of researchers (GKM, ACL, BDF, LZ, VB, RG, ND, TR, AF) screened all titles and abstracts independently. Disagreements were resolved through discussion or consulting a third senior researcher (RG) (Supplementary material D).

### Data collection

All available data from the included studies were extracted independently by rotating pairs ofresearchers (GKM, ACL, BDF, TR, ND, RB, AF), including study characteristics, design, interventions, populations, study methods, and outcomes of significance to the review question and specific objectives. Any discrepancies were resolved through discussion or consultation with a senior researcher (RG).

### Risk of bias assessment

The risk of bias was assessed independently by rotating pairs of researchers (GKM, ACL, BDF, TR, ND, RB, AF) using Version 2 of the Cochrane risk-of-bias tool for randomised trials (RoB 2).^16^ Disagreements were resolved by consensus or discussion with a senior researcher (RG).

### Grading the quality of evidence

Three authors (GKM, MH, ACL) independently assessed the certainty of evidence with Grading of Recommendations Assessment, Development, and Evaluation (GRADE).^17^ Any disagreements that arose were resolved through consensus. Four outcomes of clinical relevance, as defined in the study protocol (see later), were assessed by GRADE criteria and summarised in the corresponding evidence table.

### Outcomes

The primary outcome was the first-pass success rate, measured as a risk ratio (RR) with 95% confidence intervals (CIs). The secondary outcomes included time to achieve successful intubation; the quality of the vocal cord view, assessed using the Percentage of Glottic Opening (POGO) score^18^; the incidence of oesophageal intubation; and complications, including severe hypoxaemia, bradycardia, trauma to the airway, injury of the upper airway, and lowest oxygen saturation. Additional secondary outcomes were life-threatening events, such as cardiac arrest, failed intubation with failed ventilation, or the need for eFONA, and unplanned ICU admissions and death.

### Statistical methods

We considered only data from RCTs for the meta-analysis. With respect to effect sizes, we considered RRs for binary outcomes and mean differences (MDs) for continuous outcomes. The between-study heterogeneity was assessed with Higgins and Thompson’s *I*^2^ statistic. Given the large clinical and interstudy heterogeneity, we *a priori* chose a random-effects model to pool effect sizes as considerable between-study heterogeneity was found. For binary outcomes, the Mantel–Haenszel method was used, and for continuous outcomes, the inverse variance method. The between-study variance was based on the Paule-Mandel estimator for binary outcomes and on the restricted maximum-likelihood estimator for continuous outcomes. Associated CIs were computed with Q-Profile method and Hartung–Knapp adjustment in both cases.

When median and interquartile ranges were reported as summary statistics for continuous outcomes, a quantile estimation method was used to estimate the mean and standard deviation. A default continuity correction of 0.5 was applied in studies with zero cell frequencies. Possible publication bias was examined with Egger’s regression test (Supplementary material B).

For each outcome, we performed a subgroup analysis of the treatment effect according to the patient’s age (<1 yr and ≥1 yr) with a random-effects model without assuming a common estimate of between-study variance. All statistical computations were performed with R version 4.2.3 (R Foundation for Statistical Computing, Vienna, Austria).^19^

### Trial sequential analysis

The meta-analyses for the binary outcomes were supplemented with Trial Sequential Analysis (TSA) , combining an information size calculation for meta-analysis with a statistical significance threshold.^20^ The TSA was conducted on the primary outcome and a clinically relevant subset of the secondary outcomes. TSA was conducted with an alpha level of 5% and a beta of 80%. An O'Brien–Fleming alpha-spending function was used and heterogeneity corrections were computed with the observed *I*^2^ statistics for the corresponding outcomes. The alpha-spending boundaries are based on the observed, pooled effect sizes. Calculations were performed using TSA software version 0.9.5.10 Beta (Copenhagen Trial Unit, Copenhagen, Denmark). ^21^

## Results

We identified 22 221 articles through databases. After removing 10 706 duplicates, 11 515 study titles and abstracts were screened, and 11 309 were excluded. Of the 206 studies assessed in the full-text review, 153 were excluded. Ultimately, 53 RCTs,^11^^,^^12^^,^^22^, ^23^, ^24^, ^25^, ^26^, ^27^, ^28^, ^29^, ^30^, ^31^, ^32^, ^33^, ^34^, ^35^, ^36^, ^37^, ^38^, ^39^, ^40^, ^41^, ^42^, ^43^, ^44^, ^45^, ^46^, ^47^, ^48^, ^49^, ^50^, ^51^, ^52^, ^53^, ^54^, ^55^, ^56^, ^57^, ^58^, ^59^, ^60^, ^61^, ^62^, ^63^, ^64^, ^65^, ^66^, ^67^, ^68^, ^69^, ^70^, ^71^, ^72^ published between 2009 and 2024 and involving 4887 paediatric patients across 20 countries, met the inclusion criteria (Fig. 1). Details of the included studies are described in Supplementary Table 1.

**Fig 1:**
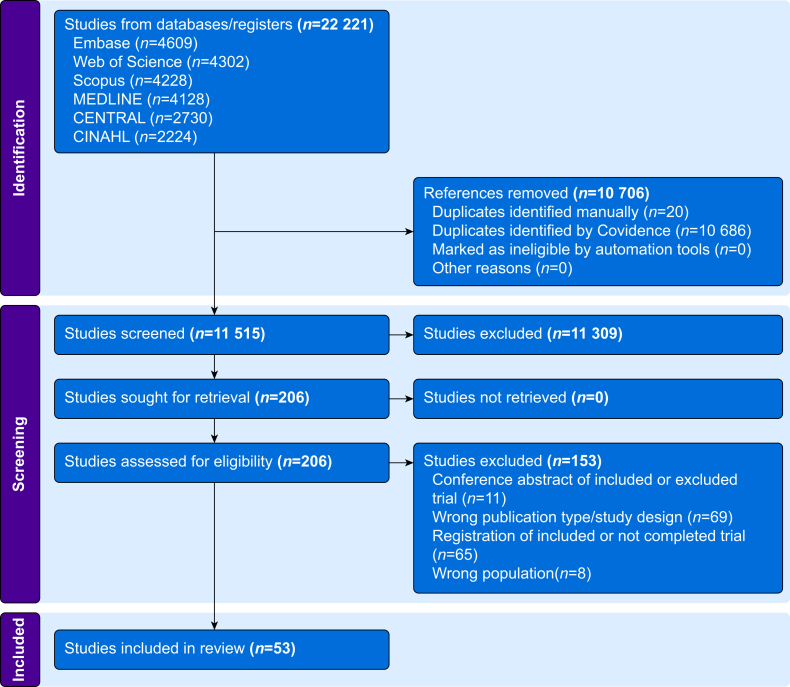
PRISMA 2020 flow diagram of study selection.

### Risk of bias assessment

Figure 2 summarises the risk of bias assessment (RoB 2). Overall, the risk of bias from RCTs was considered low in 23 trials,^11^^,^^12^^,^^22^^,^^24^, ^25^, ^26^, ^27^, ^28^, ^29^, ^30^, ^31^, ^32^, ^33^, ^34^, ^35^, ^36^, ^37^, ^38^, ^39^, ^40^, ^41^, ^42^, ^43^ moderate in 25 trials,^23^^,^^33^^,^^44^, ^45^, ^46^, ^47^, ^48^, ^49^, ^50^^,^^52^, ^53^, ^54^, ^55^, ^56^, ^57^, ^58^, ^59^, ^60^, ^61^, ^62^, ^63^, ^64^^,^^70^, ^71^, ^72^ and high in five trials.^65^, ^66^, ^67^, ^68^^,^^71^ For the randomisation process (D1), four trials^48^^,^^52^^,^^53^^,^^70^ had some concerns, whereas three trials^66^, ^67^, ^68^ were judged to have a high risk of bias because of inadequate randomisation procedures. In the domain of deviations from intended interventions (D2), seven trials^48^^,^^60^^,^^61^^,^^66^^,^^67^^,^^69^^,^^71^ showed some concerns because of insufficient details regarding the implementation of the intervention; however, none were classified as high risk. Regarding missing outcome data (D3), five trials^23^^,^^50^^,^^54^^,^^59^^,^^65^ raised some concerns, and one trial^69^ was judged to have a high risk of bias because of incomplete or inadequately reported data. For the measurement of the outcome (D4), some concerns were identified in three trials,^23^^,^^44^^,^^52^ whereas two trials^65^^,^^69^ had a high risk of bias because outcomes were defined differently between groups. Finally, for the selection of the reported result (D5), some concerns were identified in 26 trials^23^^,^^44^, ^45^, ^46^, ^47^, ^48^, ^49^^,^^51^^,^^52^^,^^55^, ^56^, ^57^, ^58^, ^59^, ^60^, ^61^, ^62^, ^63^, ^64^, ^65^, ^66^, ^67^, ^68^, ^69^^,^^71^^,^^72^ because of missing study protocols, but no trials were judged to have a high risk of bias.

**Fig 2:**
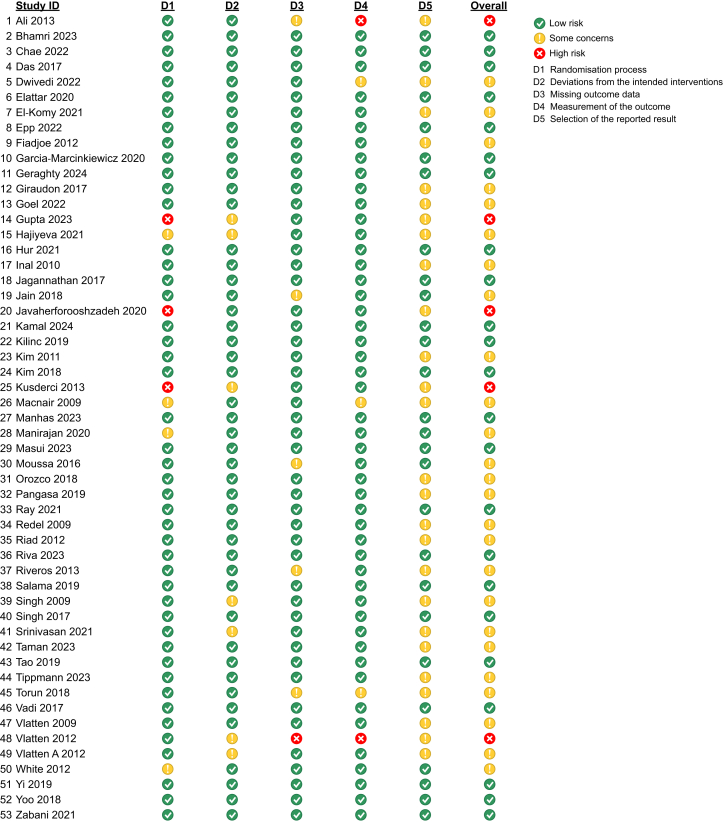
Risk of bias assessment.

### First-attempt success rate

The first-attempt tracheal intubation success rate was reported in 38 RCTs^11^^,^^12^^,^^22^^,^^23^^,^^25^^,^^27^, ^28^, ^29^, ^30^, ^31^, ^32^, ^33^, ^34^^,^^36^^,^^37^^,^^39^^,^^40^^,^^43^, ^44^, ^45^, ^46^, ^47^, ^48^, ^49^, ^50^, ^51^, ^52^, ^53^^,^^55^, ^56^, ^57^^,^^59^^,^^60^^,^^62^^,^^63^^,^^65^^,^^67^^,^^72^ including a total of 3846 patients. VL showed similar rates compared with DL, with an RR of 1.03 (95% CI: 0.99–1.07, *P*=0.18) (Fig. 3). The absolute difference corresponds to 35 more successful intubations per 1000 patients (ranging from 12 fewer to 82 more) when using VL compared with DL. The certainty of evidence was rated as high by GRADE criteria (Supplementary Table 2). The TSA showed that the cumulative Z-curve did not cross the monitoring boundary, indicating no statistically significant effect in a random-effects model with a set relative risk increase of 3%. The required information size (RIS) was estimated at 11 102 patients, but the available data accounts for only 34% of the RIS (3846/11 102), as illustrated in Supplementary Figure 1.

**Fig 3:**
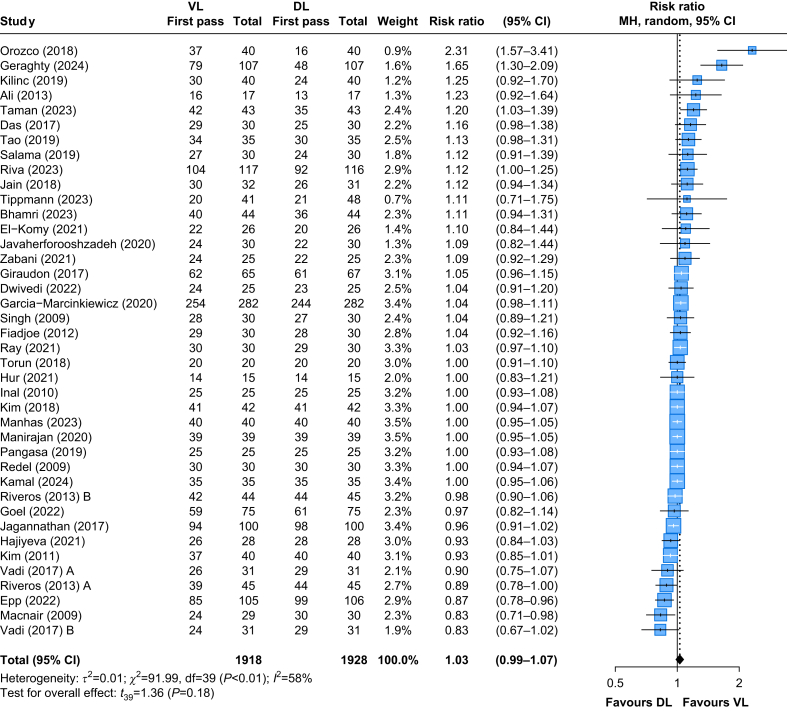
Tracheal intubation first-attempt success in paediatric patients. 95% CI, 95% confidence interval; DL, direct laryngoscopy; VL, videolaryngoscopy.

Subgroup analysis stratified by age groups revealed consistent results across both groups. Even in infants (<1 yr), VL compared with DL showed similar rates of first-attempt success (RR: 1.04, 95% CI: 0.97–1.12) and moderate heterogeneity (*I*^2^=59%). In children (≥1 yr), the results were comparable (RR: 1.02, 95% CI: 0.97–1.08), with high heterogeneity (*I*^2^=85%) (Table 1.) The test for subgroup differences was not statistically significant (*P*=0.696) (Supplementary material C—First-pass success).

**Table 1:** Subgroup analysis of RRs and MDs for major outcomes in patients <1 and ≥1 yr of age, comparing videolaryngoscopy with direct laryngoscopy. 95% CI, 95% confidence interval; MD, mean difference; POGO, Percentage of Glottic Opening; RR, risk ratio.

Outcome	Subgroup	Number of studies	RR or MD	95% CI	*τ*^2^	*I*^2^	Test for subgroup differences
First-pass success rate (%)	<1 yr	15	1.04	0.97–1.12	0.01	59.1%	*P*=0.696
	≥1 yr	25	1.02	0.97–1.08	0.01	58.0%	
Time to intubation (s)	<1 yr	17	3.5	−0.1 to 7.0	29.7	92.7%	*P*=0.653
	≥1 yr	31	2.4	−1.1 to 5.9	85.1	97.4%	
POGO (%)	<1 yr	7	12.0	2.6–21.4	80.2	86.4%	*P*=0.487
	≥1 yr	10	7.7	−3.2 to 18.6	160.0	96.3%	
Oesophageal intubation	<1 yr	2	0.16	0.1–0.4	0	0%	*P*<0.0001
	≥1 yr	5	0.94	0.3–3.1	0	0%	

### Time to intubation

The time to intubation was reported in 48 RCTs^12^^,^^22^, ^23^, ^24^, ^25^^,^^27^, ^28^, ^29^, ^30^, ^31^, ^32^, ^33^, ^34^, ^35^, ^36^, ^37^, ^38^, ^39^, ^40^, ^41^, ^42^, ^43^, ^44^, ^45^, ^46^, ^47^, ^48^, ^49^, ^50^, ^51^, ^52^, ^53^, ^54^, ^55^, ^56^, ^57^^,^^59^, ^60^, ^61^, ^62^^,^^64^, ^65^, ^66^, ^67^, ^68^, ^69^^,^^71^^,^^72^ including 4128 patients. VL was associated with 3 s longer mean time to intubation compared with DL, with an MD of 3 s (95% CI: 0.1–5.4) (Fig. 4). The certainty of evidence was rated as very low by GRADE criteria (Supplementary Table 2). The downgrading of evidence reflects very large heterogeneity across studies (*I*^2^=98%), and imprecision because of wide variations in reporting formats and study methodologies (Supplementary material C—Time to intubation).

**Fig 4:**
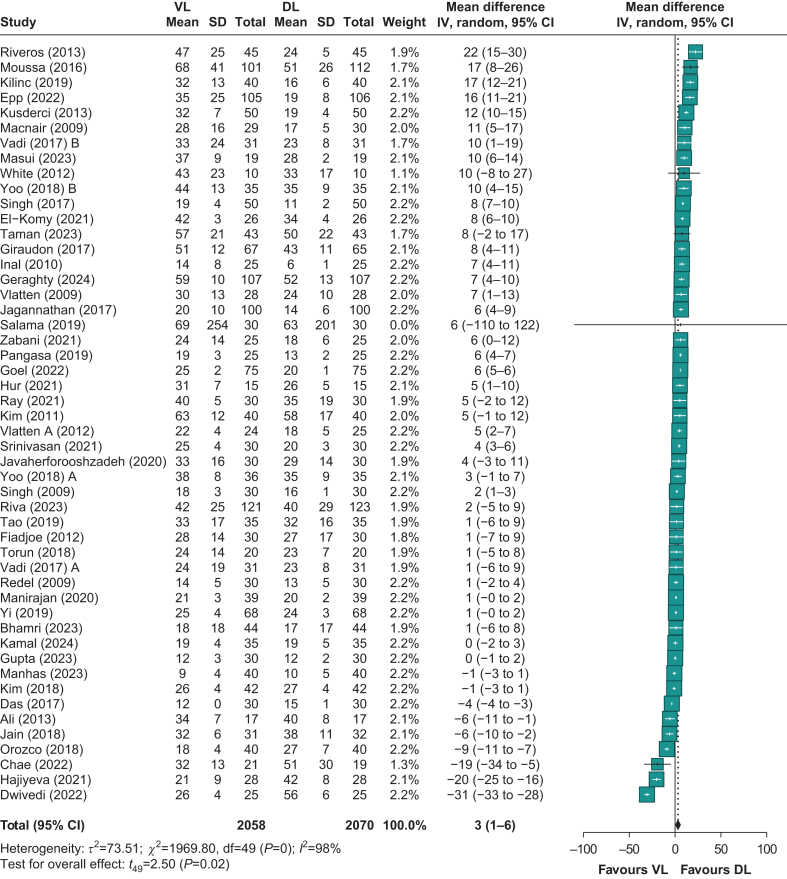
Time to intubation. 95% CI, 95% confidence interval; DL, direct laryngoscopy; VL, videolaryngoscopy.

### Percentage of Glottic Opening

The POGO score, reported in 17 RCTs^24^, ^25^, ^26^^,^^30^^,^^34^^,^^37^^,^^38^^,^^44^^,^^46^^,^^47^^,^^64^, ^65^, ^66^^,^^69^, ^70^, ^71^, ^72^ involving 1429 patients, was significantly higher with VL compared with DL. The pooled MD was 10% higher (95% CI: 3.2–16.4), favouring VL (Fig. 5). Despite this, the certainty of evidence was rated as low by GRADE criteria (Supplementary Table 2), owing to very serious inconsistency (*I*^2^=97%) across studies, which likely resulted from substantial heterogeneity in methodology, patient populations, and VL devices used.

**Fig 5:**
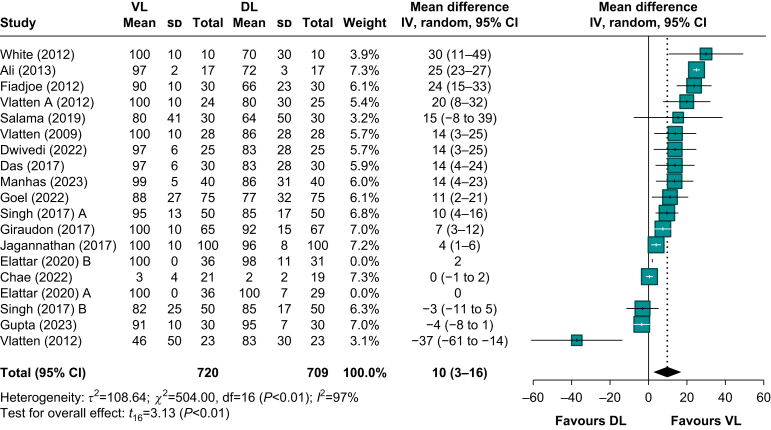
Percentage of Glottic Opening (POGO). 95% CI, 95% confidence interval; DL, direct laryngoscopy; VL, videolaryngoscopy.

### Oesophageal intubation

The rate of oesophageal intubation was reported in nine RCTs^11^^,^^25^^,^^27^^,^^35^^,^^44^^,^^46^^,^^53^^,^^63^^,^^65^ including 1431 patients. Oesophageal intubation was not different with the use of VL compared with DL (RR: 0.49, 95% CI: 0.16–1.54, *P*=0.18) (Fig. 6). This would correspond to an absolute reduction of 24 fewer oesophageal intubations per 1000 patients (ranging from seven fewer to 72 more). However, as the CI was wide, the results were not statistically significant. The certainty of evidence was rated as low by GRADE criteria, owing to imprecision and variability in point estimates across studies (Supplementary Table 2). The TSA for oesophageal intubation confirmed that the cumulative Z-curve did not cross the monitoring boundary, indicating no statistically significant effect. With a RIS of 4485 patients, the available data accounted for only 32% (1431/4485) (Supplementary Fig. 2).

**Fig 6:**
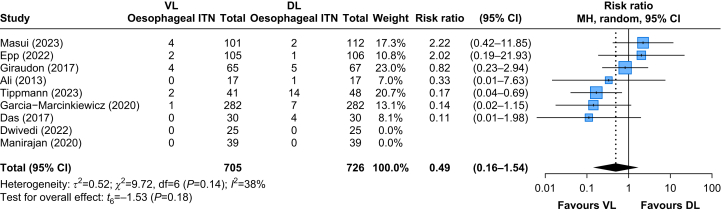
Oesophageal intubation. 95% CI, 95% confidence interval; DL, direct laryngoscopy; VL, videolaryngoscopy.

Subgroup analysis stratified by age groups revealed differences between the two groups. In infants (<1 yr), VL significantly reduced the risk of oesophageal intubation compared with DL (RR: 0.16, 95% CI: 0.1–0.4). In children (≥1 yr), the results were less pronounced (RR: 0.94, 95% CI: 0.3–3.1). The test for subgroup differences was statistically significant (*P*<0.01), suggesting that age may influence the effect of VL on the risk of oesophageal intubation (Table 1).

### Complications

Out of 53 studies, airway trauma was the most frequently reported complication in 31 RCTs.^22^^,^^24^^,^^25^^,^^27^^,^^28^^,^^31^^,^^33^^,^^36^, ^37^, ^38^, ^39^, ^40^, ^41^, ^42^, ^43^, ^44^, ^45^^,^^47^^,^^48^^,^^54^, ^55^, ^56^, ^57^^,^^59^^,^^60^^,^^63^^,^^65^^,^^68^, ^69^, ^70^^,^^72^ Hypoxaemia or desaturation was reported in 15 RCTs,^11^^,^^24^^,^^28^^,^^30^^,^^37^^,^^39^^,^^45^^,^^47^^,^^49^^,^^51^^,^^56^^,^^59^^,^^62^^,^^63^^,^^67^ whereas failed intubation was reported in 23 RCTs.^12^^,^^29^^,^^30^^,^^32^^,^^34^^,^^37^^,^^40^, ^41^, ^42^^,^^44^^,^^46^^,^^51^, ^52^, ^53^, ^54^, ^55^^,^^57^^,^^60^^,^^62^, ^63^, ^64^^,^^68^^,^^72^ Laryngospasm and bronchospasm were reported in eight RCTs.^11^^,^^12^^,^^28^^,^^31^^,^^36^^,^^46^^,^^47^^,^^53^ Haemodynamic adverse events were reported in 13 trials,^12^^,^^26^^,^^27^^,^^33^^,^^40^^,^^44^^,^^48^^,^^49^^,^^57^^,^^58^^,^^63^^,^^67^^,^^68^ and included changes in heart rate or blood pressure and episodes of bradycardia or arrhythmias; however, definitions varied, and in most cases, these changes were transient and did not require clinical intervention. Other adverse events, such as bleeding and airway manipulation, were reported in nine trials.^24^^,^^29^^,^^38^^,^^46^^,^^48^^,^^55^^,^^60^, ^61^, ^62^ Because of heterogeneous outcome definitions and variability in outcome measurement instruments across studies reporting complications, we chose not to conduct a meta-analysis. The summary table of complications is available in the Supplementary material (Supplementary Table 3).

## Discussion

This systematic review with meta-analysis compared currently available RCTs on the use of VL *vs* DL during paediatric tracheal intubation. The review analysed 53 RCTs, published between 2009 and 2024, involving 4887 paediatric patients. The primary outcome, first-attempt tracheal intubation success, resulted in no difference between VL and DL (high certainty of evidence). However, VL improved glottic visualisation across all age groups and reduced oesophageal intubation in children under 1 yr of age.

### First-attempt intubation success rate

First-attempt intubation success is an important clinical outcome, as further intubation attempts are associated with more adverse events.^7^^,^^10^^,^^73^ The meta-analysis found no statistically significant difference between VL and DL for first-attempt success. The TSA showed that the available sample size remains currently insufficient to reach statistical significance. The RIS for a conclusive result was estimated at 11 102 patients, but the available data (3846 patients) remains below this threshold. Nevertheless, first-attempt intubation success is an efficacy outcome and does not solely depend on the device used. The absence of a demonstrated difference between VL and DL in children may be influenced by methodological limitations and unaccounted confounders. Many studies had small sample sizes and were single centre, limiting generalisability. Selection bias is also a concern, as RCTs often exclude difficult airways and critically ill children who may benefit most from VL.^7^ Additionally, variations in VL devices, blade types, and operator experience with each technique introduce further confounders. Operator experience is a recognised confounder in airway management studies^12^ but was inconsistently reported across trials, limiting the ability to assess its impact on first-attempt success. The lack of reporting precluded stratified analysis and limits the generalisability of the findings to contexts involving less experienced operators. Because these factors were not explicitly described and could not be integrated into the analysis, they may have masked true differences. Larger, multicentre studies with standardised device selection, training protocols, and broader inclusion criteria are needed for a more definitive comparison. Furthermore, the variability in trial designs, outcome definitions, and measurement instruments, such as first-attempt success and complications, limits the ability to directly compare VL and DL. These considerations underscore the difficulty in conclusively determining whether VL provides a meaningful improvement over DL for first-attempt intubation success in neonates and older paediatric patients.

A consideration when first-attempt intubation success is used as primary outcome is the adequacy of a proper powered trial. For the most experienced paediatric anaesthesia setting, the chance of intubation at first attempt exceeds 95% in patients with normal airways.^10^ Lower success (about 90%) in neonates is considered as neonates are more difficult to intubate because of anatomical and physiological reasons.^11^^,^^12^ Given this already high baseline success rate, it is challenging to demonstrate superiority of VL over DL in paediatric populations. The problem in such reviews is the reliance on smaller, often single-centre trials, which are common in paediatric airway research owing to ethical and logistical constraints. As shown by the TSA, the available sample size remains insufficient to reach statistical significance contributing to the inconclusive results.

Also, this review faced the issue of selection bias in RCT on children with difficult airway as often children with relevant comorbidities are excluded.^74^ However, this population might particularly benefit from the correct application of VL to achieve first-attempt intubation success.^9^ In contrast, most children in such airway studies undergoing anaesthesia for surgical procedures are otherwise healthy without challenging airway management.

### Time to intubation

Intubation time was slightly longer with VL (MD: +3 s, 95% CI: 0.1–5.4); however, this difference is small and may not be clinically meaningful, although no universally accepted threshold exists to define clinical relevance in paediatric populations.

### Glottic visualisation

This review underlines the fact that VL enhances and magnifies the view on the glottis. This is an important advantage, especially in challenging cases. However, improved glottic visualisation does not translate into easier tracheal intubation.^13^ Especially less experienced operators might experience indirect hand–eye coordination challenges, especially in urgent situations.^75^ In time-critical emergencies, where oxygen reserves are limited, the added complexity of VL with slightly longer intubation times compared with DL calls for continuous oxygenation during airway management.^12^

### Reduced incidence of oesophageal intubation

VL appeared to reduce the incidence of oesophageal intubation in children under 1 yr of age. However, this finding is based on a limited number of studies with small sample sizes and should be considered exploratory. This potential benefit remains clinically important, as undetected oesophageal intubation can lead to severe adverse events, including death.^76^

### Limitations

A key limitation of this review is the heterogeneity in outcome definitions and measurement instruments used across the included trials, which compromises the ability to draw consistent and generalisable conclusions. This lack of standardisation hinders both meaningful comparisons between studies and the pooling of data in meta-analyses. Although failed intubation was reported as an outcome in less than half of the trials, only a few provided information on subsequent airway management. Importantly, many studies appeared to conflate difficult intubation (i.e. intubation is difficult but possible) with failed intubation (i.e. intubation is not possible), potentially leading to misclassification and limiting comparability across studies. Additionally, methodological differences, including study designs, patient selection criteria, and operator experience levels, complicate the interpretation of findings. The wide range of VL used in the studies, each with unique features and learning curves, adds another layer of inconsistency. These limitations underscore the need for future research in large multicentre studies with standardised methodologies and reporting frameworks to reduce variability and improve the reliability of evidence on the role of VL in paediatric airway management.

### Unanswered questions and future research

This finding underscores several critical unanswered questions and areas for future research. One major limitation is the significant heterogeneity across studies, driven by variations in outcome definitions and the types of VL devices evaluated. This inconsistency complicates the synthesis of evidence. To overcome this limitation, the development and implementation of a standardised paediatric airway core outcome set, including clear definitions of outcomes and outcome measurement instruments, is an important objective that is currently being addressed by an international group of paediatric anaesthesia researchers. This will facilitate the comparability of studies and enhance the quality of future research.

Additionally, specific paediatric subgroups—particularly those with difficult airways, such as those with congenital anomalies, craniofacial abnormalities, or conditions requiring urgent airway intervention—remain underrepresented in RCTs. Targeted studies focusing on these high-risk populations are essential to determine whether VL offers distinct benefits, such as reduced intubation attempts or improved first-pass success rates, in these scenarios.

Teaching laryngoscopy is an important and challenging task to prepare future airway operators. Although not included among the outcomes assessed in this systematic review, the value of VL as a teaching and training tool is widely recognised and likely contributes to its increasing adoption in clinical practice. VL enables supervisors to see exactly what the trainee sees, allowing for real-time guidance and feedback—an advantage not easily achievable with DL.^77^ Several clinical studies have demonstrated how VL improves the learning experience and facilitates skill acquisition under supervision.^54^^,^^75^^,^^76^^,^^78^^,^^79^ Nevertheless, these educational aspects were beyond the scope of our predefined outcomes and were therefore not formally evaluated.

Another crucial but underexplored factor is the impact of operator experience and the clinical setting on outcomes. The advantages of VL may depend heavily on the expertise of the user and the context in which it is used, such as emergency settings where time constraints and stress levels are heightened. Evaluating these dynamics could offer valuable insights into the training and procedural guidelines required to maximise its effectiveness. Addressing these gaps through robust, well-designed studies will be pivotal in clarifying the role of VL in paediatric airway management and optimising its application in clinical practice.

### Conclusions

This systematic review with meta-analysis and trial sequential analysis found that in paediatric patients with mostly normal airways, VL showed no statistically significant difference compared with DL in achieving first-attempt tracheal intubation success. VL improved glottic visualisation across all age groups and may offer benefits in specific subpopulations, particularly infants, by reducing the incidence of oesophageal intubation. However, the certainty of evidence for this subgroup remains low. Trial sequential analysis indicates that the available data from included studies remain underpowered to confirm a definitive effect. However, as these data were heterogeneous, more well-powered standardised RCTs are warranted with a well-defined core set of outcome variables.

## Authors’ contributions

Wrote the protocol and manuscript: GKM, ACL, MH, AA, RB, ND, JV, BDF, RG, TR, AF

Study statistician: MH

Medical librarian: JV

Articles extraction: GKM, ACL, BDF, LZ, RB, ND, TR, AF

Methodologist: AA

Data analysis and interpretation and read and approved the final manuscript: all authors

## Funding

Department of Anaesthesiology and Pain Medicine, Inselspital, Bern University Hospital, University of Bern, Bern, Switzerland.

## Declaration of interest

The authors declare that they have no conflicts of interest.

## Figures and Tables

**figs1:**
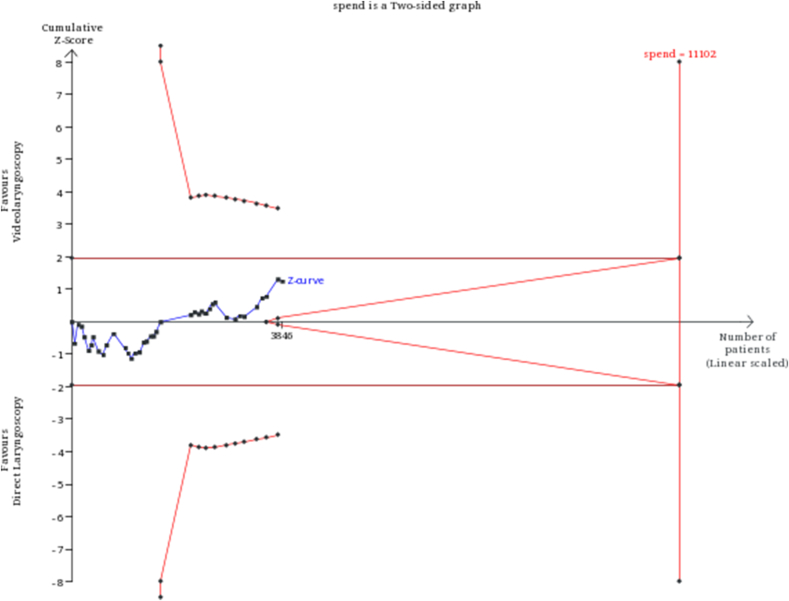


**figs2:**
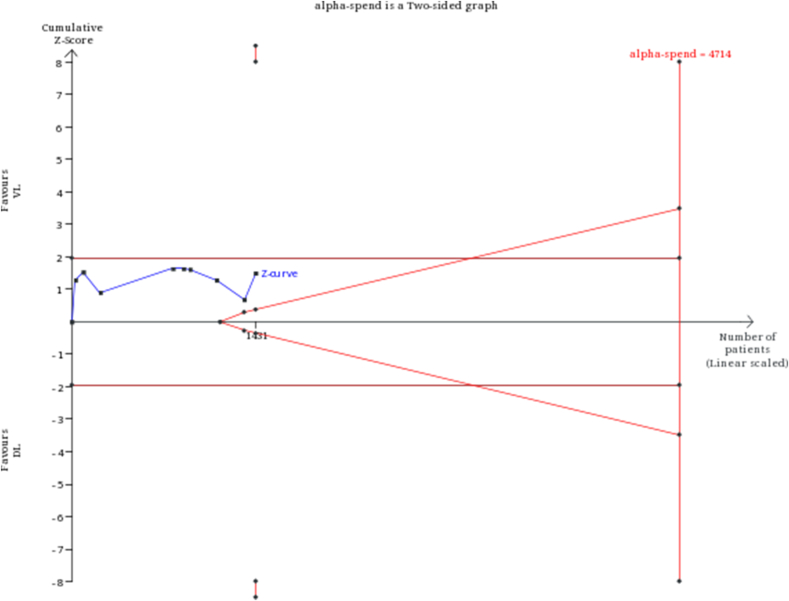


**figs3:**
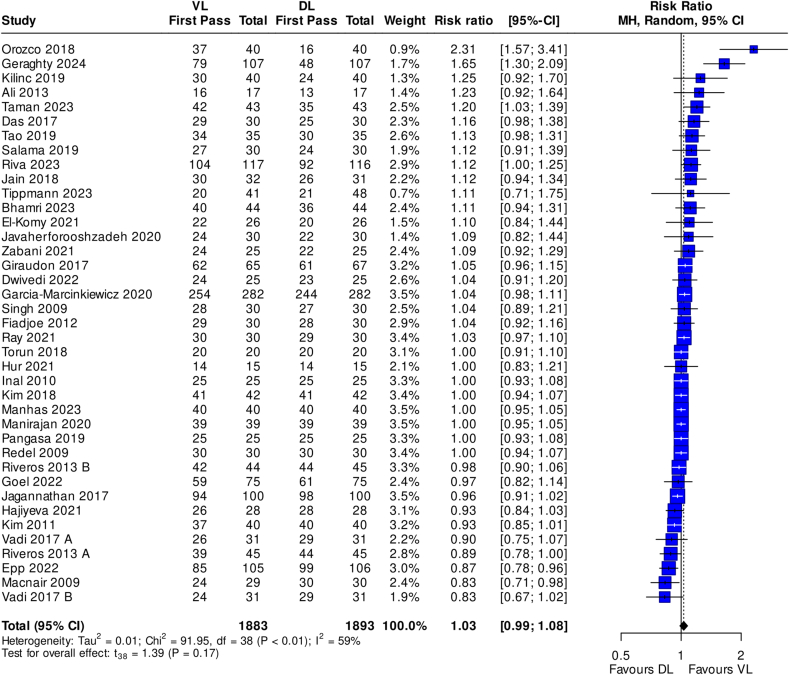


**figs4:**
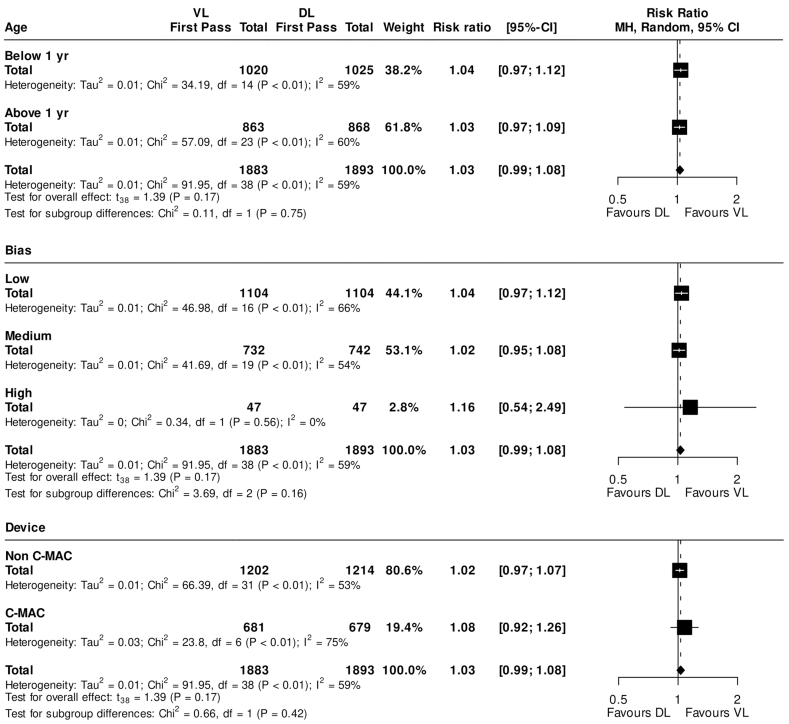


**figs5:**
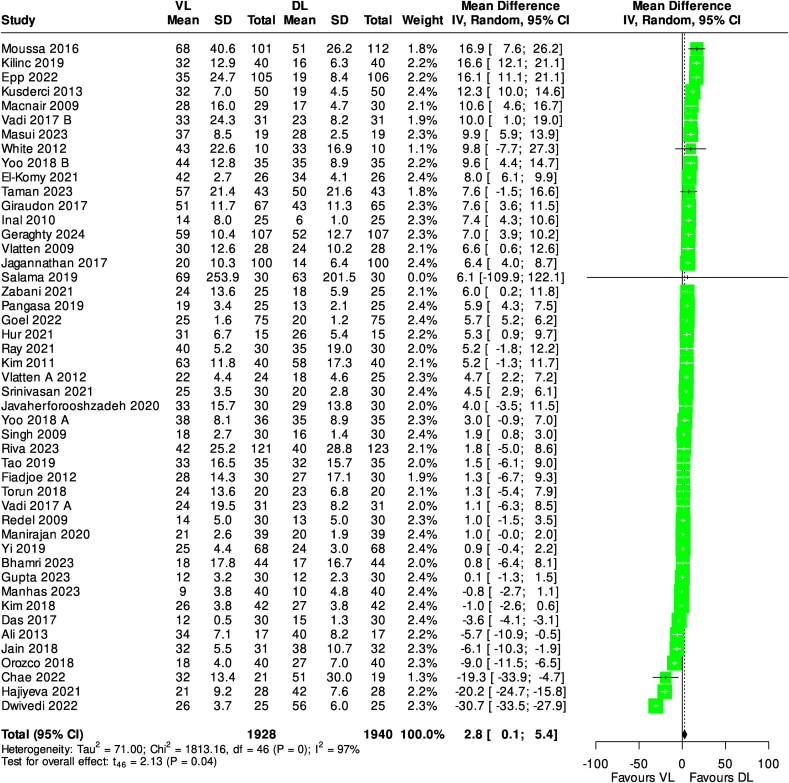


**figs6:**
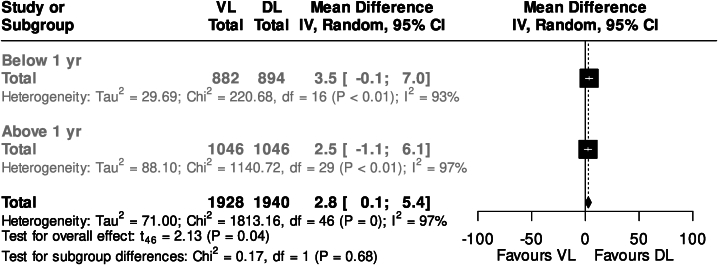


**figs7:**
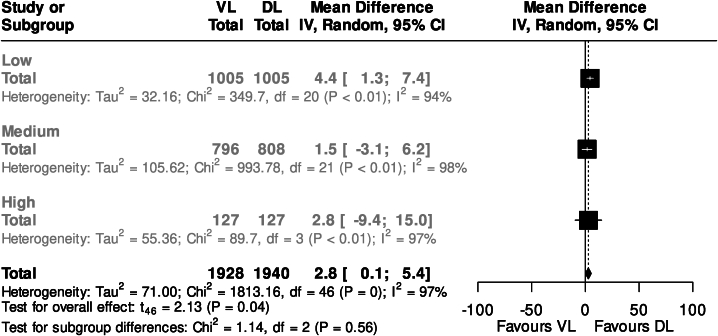


**figs8:**