# Supplementary material for: Videolaryngoscopy versus direct laryngoscopy for paediatric tracheal intubation: a systematic review with meta-analysis and trial sequential analysis
Source: Br J Anaesth. 2025 Oct 3;135(5):1486–98. doi: 10.1016/j.bja.2025.07.094 (PMC12597347; doi:10.1016/j.bja.2025.07.094)
Supplement: Multimedia Component 1 [file mmc1.docx]

**Supplemental Materials Legend**

**Supplementary Table S1.** Summary of the findings for the included studies

**Supplementary Table S2.** GRADE table of certainty of evidence for the main outcomes
**Supplementary Table S3.** Complication Summary Table

**Supplementary Figure S1.** Trial Sequential Analysis for tracheal intubation first-attempt success, showing the cumulative Z-curve and monitoring boundaries. The cumulative Z-curve did not cross the monitoring boundary, indicating no statistically significant effect in a random-effects model with a set relative risk increase of 3%. The required information size (RIS) was estimated at 11,102 patients, but the available data accounts for only 34% of the required information size (3,846/11,102).

**Supplementary Figure S2.** Trial Sequential Analysis for oesophageal intubation, showing the cumulative Z-curve and monitoring boundaries. The cumulative Z-curve did not cross the monitoring boundary, indicating no statistically significant effect. With a required information size (RIS) of 4,485 patients, the available data accounted for only 32% (1,431/4,485).

**Supplement A.** Search strategy

**Supplement B.** Egger’s regression test.

**Supplement C**. Post-hoc analyses
