# Supplementary material for: Videolaryngoscopy versus direct laryngoscopy for paediatric tracheal intubation: a systematic review with meta-analysis and trial sequential analysis
Source: Br J Anaesth. 2025 Oct 3;135(5):1486–98. doi: 10.1016/j.bja.2025.07.094 (PMC12597347; doi:10.1016/j.bja.2025.07.094)
Supplement: Multimedia Component 2 [file mmc2.docx]

### **Supplementary Table S1.** Summary of the findings for the included studies

| Author Year (Country) | Number of Patients | Setting | Age | Intervention (VL) | Control (DL) | Primary Outcome | Secondary Outcomes | Conclusions in the study | Comment |
| --- | --- | --- | --- | --- | --- | --- | --- | --- | --- |
| Ali 2013 (India) ^65^ | 34 | Operating room, single center | 1–5 years | Paediatric Airtraq | Macintosh blade | Time to intubate (TTI) (seconds) | POGO score, intubation attempts, complications: airway trauma, oesophageal intubation | Airtraq reduced TTI, improved visualization. Fewer complications with Airtraq. | Small sample size. |
| Bhamri 2023 (India)^22^ | 88 | Operating room, single center | 1–10 years | McGrath VL | Macintosh blade | Time to intubate (seconds) | Cormack-Lehane grade, glottic exposure time, first-attempt success rate, intubation difficulty scale (IDS), trauma | McGrath provided a better laryngeal view, fewer attempts, shorter glottic exposure time, and reduced trauma. | Single-center; normal paediatric airways only. |
| Chae 2022 (South Korea)^24^ | 40 | Tertiary hospital, single center | Neonates/infants | UEscope VL | Miller #0 (neonates), Macintosh #1 (infants) | Time to intubate (seconds) | Time to best view (TTBV), POGO score, Cormack-Lehane grade, desaturation rates, intraoral bleeding | UEscope reduced TTI and TTBV compared to DL, but glottic visualization was comparable. | Limited to normal paediatric airways in a single-center setting. |
| Das 2017 (India)^25^ | 60 | Operating room, single center | 2–10 years | Airtraq VL | Miller blade | Time to intubate (seconds) | POGO score, VAS for ease of intubation, oesophagealintubation, airway trauma | Airtraq improved glottic visualization and reduced trauma compared to Miller blade. | None oesophagealintubation occurred in with Airtraq. |
| Dwivedi 2022 (India)^44^ | 50 | Operating room, single center | 2–10 years | Airtraq VL | Macintosh blade | Time to intubate (seconds) | POGO score, hemodynamic parameters (BP, PR), airway trauma, intubation success rates | Airtraq showed significantly shorter TTI, better POGO score, and improved hemodynamic stability compared to DL. | Single-center study with ASA I-II children. |
| Elattar 2020 (USA)^26^ | 96 | Paediatric OR, single center | Less than 2 years | C-MAC VL | Miller blade, Wis-Hipple blade | POGO score, glottic view | Hemodynamic responses (SpO2, BP, HR), time to intubation | C-MAC provided superior glottic view compared to DL blades. Comparable hemodynamics between groups. | Focused on paediatric patients with normal airways. |
| El Komy 2021 (Egypt)^45^ | 52 | Operating room, single center | 2–6 years | Hugemed VL | Macintosh blade | Time to intubate (seconds) | Heart rate, MAP, oxygen saturation, airway trauma, number of attempts | Hugemed VL showed longer intubation time but reduced airway trauma compared to DL. Comparable hemodynamics. | Focused on beginner anesthesiologists under supervision​. |
| Epp 2022 (Germany)^27^ | 211 | Paediatric OR, multi-center | 0–12 years | King Vision aBlade VL | Macintosh blade | Success rate, learning curve | Intubation time, airway trauma, hemodynamics (HR, BP), ease of use | King Vision VL improved success rates and was easier to learn. No significant difference in airway trauma. | Focused on trainee anesthetists​​ |
| Fiadjoe 2012 (USA)^72^ | 60 | Paediatric OR, single center | Neonates/Infants | GlideScope Cobalt VL | Macintosh blade | Time to intubate (seconds) | POGO score, TTBV, airway trauma, intubation success rates | GlideScope demonstrated equivalence to DL for time to intubation and better glottic visualization. | Small sample, single-center equivalence study​​. |
| Garcia-Marcinkiewicz 2020 (USA)^11^ | 564 | Paediatric OR, multi-center | <1 year | Standard blade VL | Miller blade | First-attempt success rate | Oesophagealintubation, severe hypoxia, time to intubate, airway trauma | VL improved first-attempt success and reduced severe hypoxia compared to DL​​. | Focused on neonates and infants​. |
| Geraghty 2024 (Ireland)^28^ | 214 | NICU, single center | Neonates (<28 days) | C-MAC VL | Miller blade | First-attempt success rate | Oxygen saturation, heart rate, intubation time, oesophagealintubation | VL improved first-attempt success and reduced adverse events compared to DL​​. | Focused on neonatal intubations​. |
| Giraudon 2017 (France)^46^ | 132 | Paediatric surgery unit | 3 years (10–20 kg) | McGrath MAC VL | Macintosh DL | Time to intubate (seconds) | Time to best view (TTBV), tube passage time (TPT), glottic view (C&L grade, POGO score), adverse events | VL prolonged TTI compared to Macintosh DL without improving first attempt success or reducing adverse events. Suitable for training purposes. | Focused on novice users and teaching. |
| Goel 2022 (India)^47^ | 150 | Operating room, single center | Neonates | C-MAC VL | Miller blade | Glottic view | Intubation time, number of attempts, complications, minor trauma, desaturation, external manipulation | VL improved glottic visualization but prolonged intubation time. | Focused on neonatal intubations. |
| Gupta 2023 (India)^66^ | 60 | Operating room, single center | 2–12 years | McGrath MAC VL | Macintosh blade | POGO score | Intubation time, ease of intubation | VL provided better glottic view but had comparable intubation time. | Paediatric routine airways. |
| Hajiyeva 2021 (Turkey)^48^ | 56 | Operating room, single center | 5–10 years | C-MAC D-Blade VL | Macintosh blade | Intubation time | Attempts, hemodynamics, complications | VL significantly shortened intubation time compared to DL. | Paediatric routine airways. |
| Hur 2021 (South Korea)^29^ | 30 | Operating room, single center | 1–10 years | McGrath MAC VL | Macintosh blade | Intubation time | Glottic view, IDS, complications, Trauma | VL increased intubation time without improving glottic view. Lower IDS noted. | Children with torticollis. |
| Inal 2010 (Turkey)^49^ | 50 | Operating room, single center | 2–8 years | TruView EVO2 VL | Miller blade | Intubation Difficulty Scale (IDS) | Intubation time, glottic view (Cormack-Lehane), hemodynamic changes, lowest SpO2, success rate | TruView EVO2 provided better glottic view but required longer intubation time compared to Miller blade. | Experienced users. |
| Jagannathan 2017 (USA)^30^ | 200 | Operating room, single center | <2 years | King Vision aBlade VL | Miller blade | Intubation time | Glottic view, attempts, complications, hypoxemia, airway maneuvers required | VL provided equivalent intubation times but better glottic views. | Routine paediatric intubation cases. |
| Jain 2018 (India)^50^ | 64 | Operating room, single center | <1 year | C-MAC VL | Miller blade | Intubation difficulty | Intubation time, glottic view, success rate | VL reduced intubation difficulty in lateral positions compared to DL. | Infants intubated in lateral positions. |
| Javaherforoosh 2020 (Iran)^67^ | 60 | Paediatric cardiac surgery | 1–5 years | VL | Miller blade | Glottic view | Intubation time, desaturation, bradycardia | VL improved visualization in CHD cases but prolonged intubation time. | Congenital heart disease cases. |
|  |  | single-center) |  |  |  |  |  |  |  |
| Kamal 2024 (India)^31^ | 70 | Operating room, single center | 4–12 years | C-MAC D-blade VL | McCoy laryngoscope | Intubation difficulty scale (IDS) | Intubation success rate, Glottic view (CL grade), intubation time, time to best glottic view, laryngospasm, bronchospasm, airway trauma | VL provided better glottic views and faster time to glottic view but similar IDS and intubation times as DL. | Effective for improving glottic views in paediatric cases with normal airways; |
|  |  |  |  |  |  |  |  |  |  |
| Kilinc 2019 (Turkey)^32^ | 80 | Operating room, single center | 1–12 years | GlideScope VL | Macintosh blade | Intubation time | Glottic view, Cormack-Lehane score, VAS | VL improved glottic view but prolonged intubation time compared to DL. | Children >1 year with routine airways. |
| Kim 2011 (South Korea)^51^ | 80 | Operating room, single center | <10 years | GlideScope VL | DL (Miller/Macintosh) | Intubation time | Glottic view, complication rates, Hypoxemia, desaturation, multiple attempts | VL provided better visualization but required more attempts for similar intubation times. | Paediatric nasotracheal intubation. |
| Kim 2018 (South Korea)^33^ | 84 | Operating room, single center | 1–10 years | McGrath MAC VL | Macintosh blade | Intubation time, difficulty scale | Glottic view, success rate, hemodynamics | VL improved glottic view but had no significant advantage in intubation time. | Normal paediatric airways. |
| Kusderci 2013 (Turkey)^68^ | 100 | Operating room, single center | Neonates | TruView EVO2 VL | Miller blade | Intubation time | Glottic view, hemodynamic effects, minor airway trauma | VL prolonged intubation time compared to DL, with similar glottic views. | Neonates with routine airway |
| Macnair 2009 (UK)^52^ | 60 | Operating room, single center | 2–16 years | Berci–Kaplan VL | DL | Glottic view | Intubation time, ease of intubation | VL provided improved glottic view but prolonged intubation time. | Useful for training and elective cases. |
| Manhas 2023 (India)^34^ | 80 | Operating room, single center | 1 month–1 year | C-MAC Miller Blade VL | Miller blade | Intubation time | TBGV, POGO score, IDS, intubation success | VL reduced IDS and improved POGO score compared to DL. | Effective for routine infant intubations. |
| Manirajan 2020 (India)^53^ | 78 | Operating room, single center | <1 year | King Vision VL | Macintosh blade | Intubation time | Glottic view, first attempt success, ease of intubation | VL improved glottic view and reduced IDS, with no significant complications. | Comparable to DL for elective intubations. |
| Masui 2023 (Japan)^35^ | 50 | Operating room, single center | <24 months | McGrath MAC VL Blade 1 | Macintosh blade 1 | Intubation time | Glottic view, tube advancement difficulty, success rate | VL prolonged intubation time but provided comparable success rate. | Suitable for small children. |
| Moussa 2016 (Canada)^54^ | 40 | Operating room, single center | <6 months | GlideScope VL | Miller blade | Intubation time | Glottic view, ease of intubation, first attempt success | VL provided better glottic views but increased intubation time. | Infant airways. |
| Orozco 2018 (Venezuela)^55^ | 80 | Operating room, single center | 2–8 yrs | Airtraq VL | Macintosh blade | Intubation time | Hemodynamic response, number of attempts, airway complications | VL significantly reduced time and attempts compared to DL, with fewer complications. | Elective paediatric intubations. |
| Pangasa 2019 (India)^56^ | 50 | Operating room, single center | 2–8 yrs | Truview EVO2 VL | Macintosh blade | Time to intubation | Glottic view, intubation difficulty score, complications (airway trauma, desaturation) | VL provided better visualization but prolonged intubation time compared to DL. | Suitable for routine paediatric intubation. |
| Ray 2021 (India)^36^ | 60 | Operating room, single center | 3 months–6 yrs | Curved VL | Paraglossal DL | First-pass intubation rate | Intubation difficulty score, intubation time, airway trauma | No significant difference in success rates or IDS between VL and DL. | Focused on cleft palate surgeries. |
| Redel 2023 (Canada, Italy)^57^ | 60 | Operating room, single center | 1 month–8 yrs | GlideScope VL | Macintosh blade | Time to intubation | Glottic view, ease of intubation, hemodynamic stability | VL had no significant advantage in intubation time or complications compared to DL. | Equally effective in paediatric patients. |
| Riad 2012 (Canada)^58^ | 50 | Operating room, single center | 2–10 years | Airtraq VL | Macintosh laryngoscope | Intubation time | Number of attempts, optimization maneuvers, ease of intubation, hemodynamic changes (heart rate, BP) | Airtraq reduced intubation time, attempts, and maneuvers required; resulted in fewer hemodynamic changes compared to DL. | Effective for improving paediatric airway management in routine cases. |
|  |  |  |  |  |  |  |  |  |  |
| Riva 2023 (Multicenter)^12^ | 244 | Paediatric OR and NICU | Neonates, infants | C-MAC VL (Miller blade) | DL | First-attempt success | Desaturation, number of attempts, intubation time, adverse events | VL improved first-attempt success, reduced complications with supplemental oxygen. | Strong recommendation for VL in neonatal intubation. |
|  |  | multicentre) |  |  |  |  |  |  |  |
| Riveros 2013 (USA)^59^ | 134 | Operating room, single center | Neonates-10 yrs | Truview PCD VL, GlideScope VL | Macintosh blade | Glottic view | Time to intubation, attempts, Cormack-Lehane grade, desaturation | VLs did not improve glottic view over DL, GlideScope had worse visualization than DL. | Limited utility of VL in routine cases. |
| Salama 2019 (Egypt)^37^ | 60 | Operating room, single center | Neonates | GlideScope VL | Miller blade | POGO score | Time to best glottis view, intubation time, attempts, complications (desaturation, trauma) | VL provided better glottic views, shorter TBGV but similar intubation time compared to DL. | Lateral positioning in neonates. |
| Singh 2009 (India)^60^ | 60 | Operating room, single center | Neonates | Truview EVO2 VL | Miller blade | Glottic view | Intubation time, attempts, complications | VL improved glottic view with slightly longer intubation time compared to DL. | Neonates and infants. |
| Singh 2017 (India)^30^ | 150 | Operating room, single center | 1–6 years | Truview PCD, C-MAC VL | Macintosh blade | POGO score | Intubation time, attempts, complications | VLs provided better POGO scores but required longer intubation time compared to DL. | Focused on routine paediatric intubations. |
| Srinivasan 2021 (India)^61^ | 60 | Operating room, single center | <5 years | McGrath MAC VL | Macintosh blade | Intubation time | Glottic view, intubation success, complications | VL prolonged intubation time but reduced external laryngeal manipulation requirements. | Paediatric cleft palate surgeries. |
| Taman 2023 (Egypt)^62^ | 86 | Operating room, single center | 1–3 years | VividTrac VL | Macintosh blade | First-attempt success rate | Intubation time, attempts, complications (tube introducer use, cricoid pressure) | VL improved first-attempt success rate but increased intubation time. | Focused on cleft palate surgeries. |
| Tao 2019 (China)^39^ | 70 | Operating room, single center | Neonates | GlideScope VL | Macintosh blade | Intubation time | First-attempt success rate, glottic view, complications (desaturation) | VL improved glottic view and reduced intubation time in neonates with difficult airways (Cormack-Lehane III/IV) but not in routine intubations. | Recommended for neonates with difficult airways. |
| Tippmann 2023 (Germany)^63^ | 89 | NICU, single center | Neonates | VL | DL | First-attempt success rate | Adverse events, oesophagealintubation, desaturation, bradycardia | VL showed a trend toward reduced oesophagealintubations, but no significant improvement in first-attempt success rates. | Recommended for training neonatal intubations. |
| Çağlar Torun 2018 (Turkey)^23^ | 40 | Operating room, single center | 4–15 years | McGrath MAC VL | Macintosh blade | Intubation success | Intubation time, complications, neck extension | No significant difference between VL and DL for intubation outcomes; both effective for cerebral palsy cases. | Focused on children with cerebral palsy. |
| Vadi 2017 (USA)^40^ | 93 | Operating room, single center | <2 years | GlideScope VL, Storz VL | DL | Time to successful intubation | First-attempt success, glottic view, complications (trauma, bradycardia) | VLs improved glottic views but required longer times than DL in manual cervical spine immobilization scenarios. | Suitable for teaching trainee anesthesiologists. |
| Vlatten 2009 (Canada)^69^ | 56 | Operating room, single center | <4 years | Storz VL | DL | Time to intubation | Glottic view, ease of intubation, external manipulation | VL provided better glottic view but prolonged intubation times. | Training paediatric airway management. |
| Vlatten 2012 (Canada)^70^ | 49 | Operating room, single center | <5 years | Airtraq VL | DL | Intubation success rate | Intubation time, POGO score, external manipulation | Airtraq improved visualization but increased time to intubate and showed lower first-pass success rates. | Elective cases with experienced users. |
| Vlatten A 2012 (Canada)^64^ | 49 | Operating room, single center | 1–8 years | GlideScope VL | DL | Time to best glottic view | POGO score, laryngoscopic view, intubation attempts | VL prolonged time to best view and provided worse POGO scores compared to DL in children with cervical spine immobilization. | Immobilized cervical spines. |
| White 2012 (UK)^70^ | 60 | Operating room, multicenter | 0–6 years | Airtraq VL | DL | Intubation time | POGO score, glottic view, complications (blood staining, sore throat) | VL provided better glottic view in infants but prolonged intubation time in children compared to DL. | Elective intubations. |
| Yi 2019 (South Korea)^41^ | 136 | Operating room, single center | 1–10 years | Pentax AWS VL | Macintosh blade | Intubation time | Glottic view, success rate, complications (teeth injury, bleeding) | VL improved glottic view but did not reduce intubation time; more frequent teeth injuries noted. | Experienced practitioners. |
| Yoo 2018 (South Korea)^42^ | 108 | Operating room, single center | 1–10 years | McGrath VL, Pentax AWS | Macintosh blade | Nasotracheal intubation time | Navigation success, cuff inflation, epistaxis, complications (teeth injury) | VLs prolonged intubation times and increased cuff inflation use compared to DL, though provided clearer glottic views. | Specific nasotracheal cases. |
| Zabani 2021 (Saudi Arabia)^43^ | 50 | Operating room, single center | Neonates | GlideScope VL | Macintosh blade | Ease of tracheal intubation | Intubation time, blade insertion ease, POGO score, complications | VL achieved comparable glottic views with longer intubation time compared to DL; ease of tracheal intubation correlated with the device used. | Paediatric cardiac surgeries. |

All the studies had two arms except for studies 25,37,40,41 and 58, which had three arms.
