# Supplementary material for: Videolaryngoscopy versus direct laryngoscopy for paediatric tracheal intubation: a systematic review with meta-analysis and trial sequential analysis
Source: Br J Anaesth. 2025 Oct 3;135(5):1486–98. doi: 10.1016/j.bja.2025.07.094 (PMC12597347; doi:10.1016/j.bja.2025.07.094)
Supplement: Multimedia Component 3 [file mmc3.docx]

**Supplementary Table S2.** GRADE table of certainty of evidence for the main outcomes

|  | | | | | | |
| --- | --- | --- | --- | --- | --- | --- |
| **VL compared to DL for Tracheal intubation in children aged 0-16 years requiring elective or emergency tracheal intubation** | | | | | | |
| **Patient or population:** Tracheal intubation in children aged 0-16 years requiring elective or emergency tracheal intubation  **Setting:** in hospital patients  **Intervention: VL**  **Comparison:** DL | | | | | | |
| Outcomes | **Anticipated absolute effects^*^** (95% CI) | | Relative effect (95% CI) | № of participants (studies) | Certainty of the evidence (GRADE) | Comments |
|  | **Risk with DL** | **Risk with Videolaryngoscopy** |  |  |  |  |
| First-Pass Succes Rate ^11,12,22,23,25,27-34,36,37,39,40,43-53,55-57,59,60,62,63,65,67,72^ | 1,178 per 1,000 | **1000 per 1,000** (1,000 to 1,000) | **RR 1.03** (0.99 to 1.07) | 3,347 (38 RCTs) | ⨁⨁⨁⨁ High |  |
| Time to Intubation^12,22-25,27-57,59-62,64-69,71,72^ | The mean time to Intubation was **28.7** sec | MD **3.2 sec higher** (0.6 higher to 5.8 higher) | - | 4,128 (48 RCTs) | ⨁◯◯◯ Very low^a,b^ |  |
| Percentage of glottic opening seen (POGO) ^24-26,30,34,37,38,44,46,47,64-66,69-72^ | The mean percentage of glottic opening seen was **78.1** % | MD **10 % higher** (3 higher to 16 higher) | - | 1,429 (17 RCTs) | ⨁⨁◯◯ Low^c^ |  |
| Oesophageal intubation^11,25,27,35,44,46,53,63,65^ | 47 per 1’000 | **23 per 1,000** (7 to 72) | **RR 0.49** (0.16 to 1.54) | 1,431 (9 RCTs) | ⨁⨁◯◯ Low^d,e^ |  |
| ***The risk in the intervention group** (and its 95% confidence interval) is based on the assumed risk in the comparison group and the **relative effect** of the intervention (and its 95% CI).  **CI:** confidence interval; **MD:** mean difference; **RR:** risk ratio | | | | | | |
| **GRADE Working Group grades of evidence** **High certainty:** we are very confident that the true effect lies close to that of the estimate of the effect. **Moderate certainty:** we are moderately confident in the effect estimate: the true effect is likely to be close to the estimate of the effect, but there is a possibility that it is substantially different. **Low certainty:** our confidence in the effect estimate is limited: the true effect may be substantially different from the estimate of the effect. **Very low certainty:** we have very little confidence in the effect estimate: the true effect is likely to be substantially different from the estimate of effect. | | | | | | |

#### Explanations

a. Inconsistency: Very large heterogeneity: I-squared of 98%

b. Imprecision: Although optimal information size is likely met, there standard deviations among the studies widely differ and show large heterogeneity. In this context, the inferred estimate of the outcome was reported with substantial difference, e.g., with mean and standard deviation, with mean and 95%-confidence intervals or with median and interquartile ranges, requiring the use of transformations in order to pool the standard deviations.

c. Inconsistency: Very large heterogeneity: I-squared = 97%

d. Inconsistency: Wide variance of point estimates across studies

e. Imprecision: Optimal information size not met
