# Supplementary material for: Videolaryngoscopy versus direct laryngoscopy for paediatric tracheal intubation: a systematic review with meta-analysis and trial sequential analysis
Source: Br J Anaesth. 2025 Oct 3;135(5):1486–98. doi: 10.1016/j.bja.2025.07.094 (PMC12597347; doi:10.1016/j.bja.2025.07.094)
Supplement: Multimedia Component 5 [file mmc5.docx]

**Supplement A: Search strategies**

**Ovid MEDLINE(R) ALL <1946 to February 02, 2024>**

1 Airway Management/ 4392

2 exp Intubation, Intratracheal/ 43278

3 (airway management or respiration control or intubat*).tw,kf. 75291

4 (difficult* adj3 (airway* or visuali?ation or view)).tw,kf. 6516

5 ((difficult* or failed or cannot) adj3 (ventilat* or oxygenat* or laryngoscop*)).tw,kf. 3042

6 1 or 2 or 3 or 4 or 5 98737

7 *Laryngoscopy/ 5546

8 *Laryngoscopes/ 3215

9 Laryngoscopy/ae, co, is, mt [Adverse Effects, Complications, Instrumentation, Methods] 4838

10 Laryngoscopes/ae [Adverse Effects] 191

11 ((video* or indirect or optic* or fiberoptic*) adj5 laryngoscop*).tw,kf. 3660

12 videolaryngoscop*.tw,kf. 1659

13 (Airtraq or Pentax or King Vision or Airway Scope or Vividtrac or Res-Q-Scope or Storz or McGrath or Glidescope or ClearVue or Truview or CoPilot or UE Scope or UEScope or i-view or C-MAC or Intubrite or Anatech or Coopdech or Venner or X-lite or CEL-100 or copilot VL or uescope or Macintosh or Miller or hyperangulated blade*).mp. 13490

14 7 or 8 or 9 or 10 or 11 or 12 or 13 22887

15 6 and 14 6358

16 exp adult/ not (exp child/ or adolescent/ or exp infant/ or exp Pediatrics/) 6174883

17 15 not 16 4249

18 exp animals/ not humans.sh. 5193800

19 17 not 18 4223

20 limit 19 to yr="2000 - 2024" 3666

**Embase <1974 to 2024 February 02>**

1 respiration control/ 21499

2 exp endotracheal intubation/ 64701

3 exp difficult airway management/ 1425

4 (airway management or respiration control or intubat*).tw,kf. 122684

5 (difficult* adj3 (airway* or visuali?ation or view)).tw,kf. 10054

6 ((difficult* or failed or cannot) adj3 (ventilat* or oxygenat* or laryngoscop*)).tw,kf. 4582

7 1 or 2 or 3 or 4 or 5 or 6 169043

8 videolaryngoscopy/ 1470

9 videolaryngoscope/ 4030

10 inDL/ 103

11 ((video* or indirect or optic* or fiberoptic*) adj5 laryngoscop*).tw,kf. 5209

12 videolaryngoscop*.tw,kf. 2459

13 (Airtraq or Pentax or King Vision or Airway Scope or Vividtrac or Res-Q-Scope or Storz or McGrath or Glidescope or ClearVue or Truview or CoPilot or UE Scope or UEScope or i-view or C-MAC or Intubrite or Anatech or Coopdech or Venner or X-lite or CEL-100 or copilot VL or uescope or Macintosh or Miller or hyperangulated blade*).mp. 30081

14 8 or 9 or 10 or 11 or 12 or 13 35612

15 7 and 14 8063

16 exp adult/ not (exp child/ or exp adolescent/ or exp infant/ or exp Pediatrics/) 9547373

17 15 not 16 4482

18 (animal/ or Animal experiment/) not (human experiment/ or human/) 3802415

19 17 not 18 4452

20 limit 19 to yr="2000 - 2025" 4251

[**Cochrane Central Register of Controlled Trials**](https://www.cochranelibrary.com/)
Issue 2 of 12, February 2024

#1 MeSH descriptor: [Airway Management] explode all trees 14311

#2 MeSH descriptor: [Intubation, Intratracheal] explode all trees 5703

#3 ("airway management" or "respiration control" or intubat*):ti,ab,kw 26507

#4 (difficult* NEAR/3 (airway* or visuali*ation or view)):ti,ab,kw 1523

#5 ((difficult* or failed or cannot) NEAR/3 (ventilat* or oxygenat* or laryngoscop*)):ti,ab,kw 716

#6 #1 or #2 or #3 or #4 or #5 34743

#7 MeSH descriptor: [Laryngoscopy] explode all trees 1421

#8 MeSH descriptor: [Laryngoscopes] explode all trees 818

#9 ((video* or indirect or optic* or fiberoptic*) NEAR/5 laryngoscop*):ti,ab,kw 1690

#10 (videolaryngoscop*):ti,ab,kw 1246

#11 (Airtraq or Pentax or King Vision or Airway Scope or Vividtrac or Res-Q-Scope or Storz or McGrath or Glidescope or ClearVue or Truview or CoPilot or UE Scope or UEScope or i-view or C-MAC or Intubrite or Anatech or Coopdech or Venner or X-lite or CEL-100 or copilot VL or uescope or Macintosh or Miller or hyperangulated blade*):ti,ab,kw 4163

#12 #7 or #8 or #9 or #10 or #11 5632

#13 #6 and #12 3415

#14 MeSH descriptor: [Adult] explode all trees 611867

#15 MeSH descriptor: [Child] explode all trees 81197

#16 MeSH descriptor: [Infant] explode all trees 45750

#17 MeSH descriptor: [Pediatrics] explode all trees 1042

#18 MeSH descriptor: [Adolescent] explode all trees 136261

#19 #15 or #16 or #17 or #18 203713

#20 #14 not #19 495239

#21 #13 not #20 with Cochrane Library publication date Between Jan 2000 and Dec 2024, in Trials 2435

**CINAHL (Ebsco)**

| S1 | (MH "Airway Management+") OR (MH "Intubation, Intratracheal+") |  |  | 25,558 |
| --- | --- | --- | --- | --- |
| S2 | TI ( (airway management or respiration control or intubat*) ) OR AB ( (airway management or respiration control or intubat*) ) |  |  | 23,416 |
| S3 | TI ( (difficult* N3 (airway* or visuali#ation or view)) ) OR AB ( (difficult* N3 (airway* or visuali#ation or view)) ) |  |  | 2,282 |
| S4 | TI ( ((difficult* or failed or cannot) N3 (ventilat* or oxygenat* or laryngoscop*)) ) OR AB ( ((difficult* or failed or cannot) N3 (ventilat* or oxygenat* or laryngoscop*)) ) |  |  | 1,230 |
| S5 | S1 OR S2 OR S3 OR S4 |  |  | 39,435 |
| S6 | (MH "Laryngoscopy") |  |  | 5,684 |
| S7 | TI ( ((video* or indirect or optic* or fiberoptic*) N5 laryngoscop*) ) OR AB ( ((video* or indirect or optic* or fiberoptic*) N5 laryngoscop*) ) |  |  | 1,343 |
| S8 | TI videolaryngoscop* OR AB videolaryngoscop* |  |  | 732 |
| S9 | TI ( Airtraq or Pentax or King Vision or Airway Scope or Vividtrac or Res-Q-Scope or Storz or McGrath or Glidescope or ClearVue or Truview or CoPilot or UE Scope or UEScope or i-view or C-MAC or Intubrite or Anatech or Coopdech or Venner or X-lite or CEL-100 or copilot VL or uescope or Macintosh or Miller or hyperangulated blade*) ) OR AB ( Airtraq or Pentax or King Vision or Airway Scope or Vividtrac or Res-Q-Scope or Storz or McGrath or Glidescope or ClearVue or Truview or CoPilot or UE Scope or UEScope or i-view or C-MAC or Intubrite or Anatech or Coopdech or Venner or X-lite or CEL-100 or copilot VL or uescope or Macintosh or Miller or hyperangulated blade*) ) |  |  | 4,861 |
| S10 | S6 OR S7 OR S8 OR S9 |  |  | 10,398 |
| S11 | S5 AND S10 |  |  | 3,473 |
| S12 | (MH "Adult+") |  |  | 2,095,805 |
| S13 | (MH "Child+") |  |  | 769,815 |
| S14 | (MH "Pediatrics+") |  |  | 23,340 |
| S15 | (MH "Adolescence+") |  |  | 614,345 |
| S16 | S13 OR S14 OR S15 |  |  | 1,141,572 |
| S17 | S12 NOT S16 |  |  | 1,661,817 |
| S18 | S11 NOT S17 |  |  | 2,303 |
| S19 | S11 NOT S17 Date: 20000101-20241231 |  |  | 2,117 |

**Web of Science Core Collection**

1 TS=((difficult* NEAR/3 (airway* or visualization or visualisation or view)) or ((difficult* or failed or cannot) NEAR/3 (ventilat* or oxygenat* or laryngoscop*)) or “airway management” or “respiration control” or intubat*) [81,055](https://www-webofscience-com.ep.fjernadgang.kb.dk/wos/woscc/summary/78844c85-ba31-4292-84f2-03c28f180d4e-c9c1d479/relevance/1)

2 TS=(((video* or indirect or optic* or fiberoptic*) NEAR/5 laryngoscop*) or (videolaryngoscop*) or (Airtraq or Pentax or King Vision or Airway Scope or Vividtrac or Res-Q-Scope or Storz or McGrath or Glidescope or ClearVue or Truview or CoPilot or UE Scope or UEScope or i-view or C-MAC or Intubrite or Anatech or Coopdech or Venner or X-lite or CEL-100 or copilot VL or uescope or Macintosh or Miller or hyperangulated blade*)) [49,664](https://www-webofscience-com.ep.fjernadgang.kb.dk/wos/woscc/summary/528e5a4f-b833-4c86-bb1d-9f6e225df40a-c9c1d775/relevance/1)

3 #2 AND #1 [4,703](https://www-webofscience-com.ep.fjernadgang.kb.dk/wos/woscc/summary/f5b6f309-e4a6-4b51-91e6-e48f4e317307-c9c217de/relevance/1)

4 TS=((adult* not (child* OR infant* OR neonat* OR paediatric* or pediatric* or young or youth or adolescen* or newborn))) [1,435,195](https://www-webofscience-com.ep.fjernadgang.kb.dk/wos/woscc/summary/db32ccd3-8787-41e0-9802-98dd241fed7f-c9c21bba/relevance/1)

5 #3 NOT #4 [4,177](https://www-webofscience-com.ep.fjernadgang.kb.dk/wos/woscc/summary/163cf379-472a-4cde-99fd-09d0469dbe9c-c9c221d6/relevance/1)

6 #3 NOT #4 Date: 20000101-20241231 [4,009](https://www-webofscience-com.ep.fjernadgang.kb.dk/wos/woscc/summary/c338a1e7-5f58-4c5e-9993-00cc9f12beb6-c9c22c3d/relevance/1)

SCOPUS

1
TITLE-ABS-KEY ( "airway management" OR "respiration control" OR intubat* OR ( ( difficult* OR failed OR cannot ) W/3 ( ventilat* OR oxygenat* OR laryngoscop* ) ) OR ( difficult* W/3 ( airway* OR visuali*ation OR view ) ) )
[174,705 results](https://www-scopus-com.ep.fjernadgang.kb.dk/search/history/results.uri?origin=searchhistory&shid=5)

2
TITLE-ABS-KEY ( ( ( video* OR indirect OR optic* OR fiberoptic* ) W/5 laryngoscop* ) OR videolaryngoscop* OR airtraq OR pentax OR "king vision" OR "airway scope" OR vividtrac OR res-q-scope OR storz OR mcgrath OR glidescope OR clearvue OR truview OR copilot OR "ue scope" OR uescope OR i-view OR c-mac OR intubrite OR anatech OR coopdech OR venner OR x-lite OR cel-100 OR "copilot vl" OR uescope OR macintosh OR miller OR ( hyperangulated W/2 blade* ) )
[54,074 results](https://www-scopus-com.ep.fjernadgang.kb.dk/search/history/results.uri?origin=searchhistory&shid=6)

3
( TITLE-ABS-KEY ( ( ( video* OR indirect OR optic* OR fiberoptic* ) W/5 laryngoscop* ) OR videolaryngoscop* OR airtraq OR pentax OR "king vision" OR "airway scope" OR vividtrac OR res-q-scope OR storz OR mcgrath OR glidescope OR clearvue OR truview OR copilot OR "ue scope" OR uescope OR i-view OR c-mac OR intubrite OR anatech OR coopdech OR venner OR x-lite OR cel-100 OR "copilot vl" OR uescope OR macintosh OR miller OR ( hyperangulated W/2 blade* ) ) ) AND ( TITLE-ABS-KEY ( "airway management" OR "respiration control" OR intubat* OR ( ( difficult* OR failed OR cannot ) W/3 ( ventilat* OR oxygenat* OR laryngoscop* ) ) OR ( difficult* W/3 ( airway* OR visuali*ation OR view ) ) ) )[6,121 results](https://www-scopus-com.ep.fjernadgang.kb.dk/search/history/results.uri?origin=searchhistory&shid=7)

4
TITLE-ABS-KEY ( adult* AND NOT ( child* OR infant* OR neonat* OR paediatric* or pediatric* or young or youth or adolescen* ) )
[6,695,753 results](https://www-scopus-com.ep.fjernadgang.kb.dk/search/history/results.uri?origin=searchhistory&shid=8)

5
( ( TITLE-ABS-KEY ( ( ( video* OR indirect OR optic* OR fiberoptic* ) W/5 laryngoscop* ) OR videolaryngoscop* OR airtraq OR pentax OR "king vision" OR "airway scope" OR vividtrac OR res-q-scope OR storz OR mcgrath OR glidescope OR clearvue OR truview OR copilot OR "ue scope" OR uescope OR i-view OR c-mac OR intubrite OR anatech OR coopdech OR venner OR x-lite OR cel-100 OR "copilot vl" OR uescope OR macintosh OR miller OR ( hyperangulated W/2 blade* ) ) ) AND ( TITLE-ABS-KEY ( "airway management" OR "respiration control" OR intubat* OR ( ( difficult* OR failed OR cannot ) W/3 ( ventilat* OR oxygenat* OR laryngoscop* ) ) OR ( difficult* W/3 ( airway* OR visuali*ation OR view ) ) ) ) ) AND NOT ( TITLE-ABS-KEY ( adult* AND NOT ( child* OR infant* OR neonat* OR paediatric* or pediatric* or young or youth or adolescen* ) ) )
[4,172 results](https://www-scopus-com.ep.fjernadgang.kb.dk/search/history/results.uri?origin=searchhistory&shid=9)

6
( ( TITLE-ABS-KEY ( ( ( video* OR indirect OR optic* OR fiberoptic* ) W/5 laryngoscop* ) OR videolaryngoscop* OR airtraq OR pentax OR "king vision" OR "airway scope" OR vividtrac OR res-q-scope OR storz OR mcgrath OR glidescope OR clearvue OR truview OR copilot OR "ue scope" OR uescope OR i-view OR c-mac OR intubrite OR anatech OR coopdech OR venner OR x-lite OR cel-100 OR "copilot vl" OR uescope OR macintosh OR miller OR ( hyperangulated W/2 blade* ) ) ) AND ( TITLE-ABS-KEY ( "airway management" OR "respiration control" OR intubat* OR ( ( difficult* OR failed OR cannot ) W/3 ( ventilat* OR oxygenat* OR laryngoscop* ) ) OR ( difficult* W/3 ( airway* OR visuali*ation OR view ) ) ) ) ) AND NOT ( TITLE-ABS-KEY ( adult* AND NOT ( child* OR infant* OR neonat* OR paediatric* or pediatric* or young or youth or adolescen* ) ) ) AND ( LIMIT-TO ( PUBYEAR , 2000 ) OR LIMIT-TO ( PUBYEAR , 2001 ) OR LIMIT-TO ( PUBYEAR , 2002 ) OR LIMIT-TO ( PUBYEAR , 2003 ) OR LIMIT-TO ( PUBYEAR , 2004 ) OR LIMIT-TO ( PUBYEAR , 2005 ) OR LIMIT-TO ( PUBYEAR , 2006 ) OR LIMIT-TO ( PUBYEAR , 2007 ) OR LIMIT-TO ( PUBYEAR , 2008 ) OR LIMIT-TO ( PUBYEAR , 2009 ) OR LIMIT-TO ( PUBYEAR , 2010 ) OR LIMIT-TO ( PUBYEAR , 2011 ) OR LIMIT-TO ( PUBYEAR , 2012 ) OR LIMIT-TO ( PUBYEAR , 2013 ) OR LIMIT-TO ( PUBYEAR , 2014 ) OR LIMIT-TO ( PUBYEAR , 2015 ) OR LIMIT-TO ( PUBYEAR , 2016 ) OR LIMIT-TO ( PUBYEAR , 2017 ) OR LIMIT-TO ( PUBYEAR , 2018 ) OR LIMIT-TO ( PUBYEAR , 2019 ) OR LIMIT-TO ( PUBYEAR , 2020 ) OR LIMIT-TO ( PUBYEAR , 2021 ) OR LIMIT-TO ( PUBYEAR , 2022 ) OR LIMIT-TO ( PUBYEAR , 2023 ) OR LIMIT-TO ( PUBYEAR , 2024 ) )
[3,911 results](https://www-scopus-com.ep.fjernadgang.kb.dk/search/history/results.uri?origin=searchhistory&shid=31)
