# Supplementary material for: Videolaryngoscopy versus direct laryngoscopy for paediatric tracheal intubation: a systematic review with meta-analysis and trial sequential analysis
Source: Br J Anaesth. 2025 Oct 3;135(5):1486–98. doi: 10.1016/j.bja.2025.07.094 (PMC12597347; doi:10.1016/j.bja.2025.07.094)
Supplement: Multimedia Component 6 [file mmc6.docx]

**Supplement B.** Egger’s regression test.

|  | Intercept | 95%-CI | t-statistic | p-value |
| --- | --- | --- | --- | --- |
| **First pass success rate (%)** | 0.16 | 0.24 to 2.08 | 2.478 | 0.018 |
| **Time to intubation (sec)** | 0.59 | -1.83 to 3.01 | 0.48 | 0.633 |
| **POGO (%)** | -0.71 | 4.81 to 3.40 | -0.337 | 0.741 |
| **Oesophageal intubation** | -0.65 | -4.01 to 2.71 | -0.38 | 0.720  (meta-analysis contains k = 7 studies. Egger's test may lack the statistical power to detect bias when the number of studies is small (i.e., k<10)) |
